# Pol B, a Family B DNA Polymerase, in *Thermococcus kodakarensis* is Important for DNA Repair, but not DNA Replication

**DOI:** 10.1264/jsme2.ME19075

**Published:** 2019-07-27

**Authors:** Takashi Kushida, Issay Narumi, Sonoko Ishino, Yoshizumi Ishino, Shinsuke Fujiwara, Tadayuki Imanaka, Hiroki Higashibata

**Affiliations:** 1 Graduate School of Life Sciences, Toyo University 1–1–1 Izumino, Itakura-machi, Ora-gun, Gunma 374–0193 Japan; 2 Department of Bioscience and Biotechnology, Graduate School of Bioresource and Bioenvironmental Sciences, Kyushu University 744 Motooka, Nishi-ku, Fukuoka 819–0395 Japan; 3 Department of Bioscience, School of Science and Technology, Kwansei-Gakuin University 2–1 Gakuen, Sanda, Hyogo 669–1337 Japan; 4 Research Organization of Science and Technology, Ritsumeikan University 1–1–1 Noji-higashi, Kusatsu, Shiga 525–8577 Japan

**Keywords:** family B DNA polymerase, DNA repair, DNA replication hyperthermophilic archaea

## Abstract

*Thermococcus kodakarensis* possesses two DNA polymerases, Pol B and Pol D. We generated a *T. kodakarensis* strain (DPB1) in which *polB* was completely deleted and a derivative of DPB1 in which *polB* was overexpressed; neither of the generated strains exhibited any growth delay, indicating that the lack or overexpression of Pol B in *T. kodakarensis* did not affect cell growth. We also found that DPB1 showed higher sensitivity to four DNA-damaging agents (ultraviolet C irradiation, γ-ray irradiation, methyl methanesulfonate, and mitomycin C) than the parental strain. The sensitivity of DPB1 was restored to the level of the parent strain by the introduction of a plasmid harboring *polB*, suggesting that the DNA damage-sensitive phenotype of DPB1 was due to the loss of *polB*. Collectively, these results indicate that Pol B is involved in DNA repair, but not DNA replication, which, in turn, implies that Pol D is the sole replicative DNA polymerase in *Thermococcus* species.

DNA polymerases are enzymes that synthesize complementary DNA strands of template DNA and, thus, are important for DNA replication and repair. Most living organisms possess several DNA polymerases, which are classified into the following 7 families based on their primary structures: A, B, C, D, E, X, and Y (2, 5, 24, 30).

*Archaea*, constituting the third domain of life, are useful for elucidating the mechanisms involved in the maintenance and transfer of genetic information, particularly from an evolutionary point of view. The study of archaeal DNA replication started in the 1980s, and once it was found that aphidicolin, a specific inhibitor of family B DNA polymerases, inhibits DNA synthesis and cell growth in halophilic and methanogenic archaea, it became apparent that DNA replication in archaea differs from that in bacteria (7, 46). This also suggested that archaeal DNA polymerases resemble the eukaryotic DNA polymerases Pols α, δ, and ɛ because these family B DNA polymerases were inhibited by aphidicolin. The amino acid sequences deduced from cloned genes from three different archaea (*Thermococcus litoralis*, *Sulfolobus solfataricus*, and *Pyrococcus furiosus*) showed sequence similarities with those of the family B DNA polymerases (31, 32, 41). Examinations of the first archaeal genome to be fully sequenced, that of *Methanocaldococcus jannaschii*, led to the conclusion that, in contrast to eukaryotes, archaea contain only a single Pol B homologue (3), even though two different family B DNA polymerases had been reported earlier in *Pyrodictium occultum* (42).

In a study to identify new DNA polymerases in *P. furiosus*, Uemori *et al*. constructed a genomic library that was expressed in *Escherichia coli* and then screened the expressed proteins for polymerase activity at a high temperature, which led to the discovery of the family D DNA polymerase Pol D (43). Pol D is an archaeal-specific DNA polymerase that does not have amino acid sequence similarity with any other DNA polymerase (5). Total genome sequences have shown that all archaeal species, except *Crenarchaeota*, possess both Pol B and Pol D. The biochemical properties of these enzymes, such as stimulation by proliferation cell nuclear antigen (PCNA) and 3′ → 5′ exonuclease proofreading activity, are consistent with those of replicative polymerases (18, 39). Knockout experiments on the genes encoding Pol B and Pol D in *Halobacterium* NRC-1 suggested that both DNA polymerases are involved in DNA replication and are essential for cell viability (1). *In vitro* biochemical characterization, such as primer usage and strand-displacement activity (16, 17), have suggested that Pol B replicates the leading strand, whereas Pol D replicates the lagging strand. This is similar to the activities of Pol ɛ, which replicates the leading strand, and Pol δ, which replicates the lagging strand, in eukaryotes, showing that the systems of DNA replication in archaea and eukaryotes are similar.

*Thermococcales* members are the most frequently isolated hyperthermophiles. They are widely distributed at deep-sea and shallow marine hydrothermal vents as well as terrestrial thermal springs. At elevated temperatures, spontaneous DNA reactions, such as the hydrolytic deamination of cytosines and adenines, hydrolytic depurination, the oxidation of guanines, methylation of bases and phosphates, and strand breakage, are accelerated (23). Since hyperthermophiles inhabit such extremely hostile conditions, they possess stress adaptation mechanisms to ensure recovery from heat damage. *T. kodakarensis*, belonging to *Thermococcales*, has been isolated from a solfatara on the shore of Kodakara Island, Japan (26). Genetic manipulation methods have been developed for *T. kodakarensis*. *E. coli*–*T. kodakarensis* shuttle vectors and strong constitutive promoters for gene expression may be used (21). In the present study, we generated a *T. kodakarensis* strain in which *polB* was completely deleted and investigated the effects of exposure to DNA-damaging agents on this mutant. Our results indicate that Pol B is involved in DNA repair, not DNA replication, in *T. kodakarensis*.

## Materials and Methods

### Strains, media, and plasmids

*T. kodakarensis* strain DAD (genotype, Δ*pyrF* Δ*pdaD*) was used as the parental strain (9). Strains were cultivated in a nutrient-rich medium (ASW-YT-S^0^), synthetic medium (ASW-AA-S^0^ medium), or medium for Southern and Western blotting (ASW-YT-Pyr) under strict anaerobic conditions at 85°C (37). ASW-YT-S^0^ medium contained 0.8×artificial seawater (ASW), 5 g L^−1^ yeast extract, 5 g L^−1^ tryptone, and 2 g L^−1^ elemental sulfur (S^0^). ASW-AA-S^0^ medium contained 0.8×ASW, a mixture of 20 amino acids, modified Wolfe’s trace minerals, a vitamin mixture, and 2 g L^−1^ elemental sulfur (34, 37). ASW-YT-Pyr medium contained 0.8×ASW, 5 g L^−1^ yeast extract, 5 g L^−1^ tryptone, and 5 g L^−1^ sodium pyruvate. Resazurin sodium salt, a redox indicator, was added to ASW-YT-S^0^, ASW-AA-S^0^, and ASW-YT-Pyr media at concentrations of 2, 1, and 2 mg L^−1^, respectively. Na_2_S solution (5%) was added to ASW-YT-S^0^, ASW-AA-S^0^, and ASW-YT-Pyr media at concentrations of 0.025, 0.0075, and 0.025%, respectively, to make the medium color transparent prior to the inoculation. To prepare solid ASW-YT media, 2 mL L^−1^ polysulfide solution (10 g of Na_2_S·9H_2_O and 3 g of elemental sulfur in 15 mL of H_2_O) instead of S^0^ and 1% gellan gum were added to liquid media (27).

*E. coli* strain DH5α and the plasmid pUC19 (Takara Bio, Kyoto, Japan) were used for DNA manipulation. *E. coli* cells harboring pUC19 or its derivatives were cultured at 37°C in 2×YT medium (16 g L^−1^ tryptone, 10 g L^−1^ yeast extract, and 5 g L^−1^ NaCl) supplemented with 50 mg L^−1^ ampicillin.

The plasmid pUD3 containing a *pyrF* marker gene cassette was used for targeted gene disruption (45). The *E. coli*–*T. kodakarensis* shuttle plasmid pTKG02, a derivative of pTK02 previously reported by Nagaoka *et al.* (27), was used for gene complementation.

Unless otherwise mentioned, all chemicals were purchased from FUJIFILM Wako Pure Chemical Corporation (Osaka, Japan), Sigma-Aldrich (St. Louis, MO, USA), or Nacalai Tesque (Kyoto, Japan).

### DNA manipulation

DNA manipulations were performed according to standard methods, as previously described by Sambrook and Russell (35). Restriction endonucleases and modification enzymes were purchased from Takara Bio and Toyobo (Osaka, Japan). DNA amplifications were performed with KOD-Plus- ver. 2 DNA polymerase (Toyobo) and the oligonucleotide primers listed in Table 1. The extraction and purification of plasmids from *E. coli* and of DNA fragments from agarose gels were performed with a QIAprep Spin Miniprep Kit and QIAquick Gel Extraction Kit, respectively (QIAGEN, Hilden, Germany). The DNA sequences of all plasmids constructed in the present study were confirmed by DNA sequencing performed on our behalf by Eurofins Genomics (Tokyo, Japan). *Disruption of polB in T. kodakarensis DAD*

**Fig. 1:**
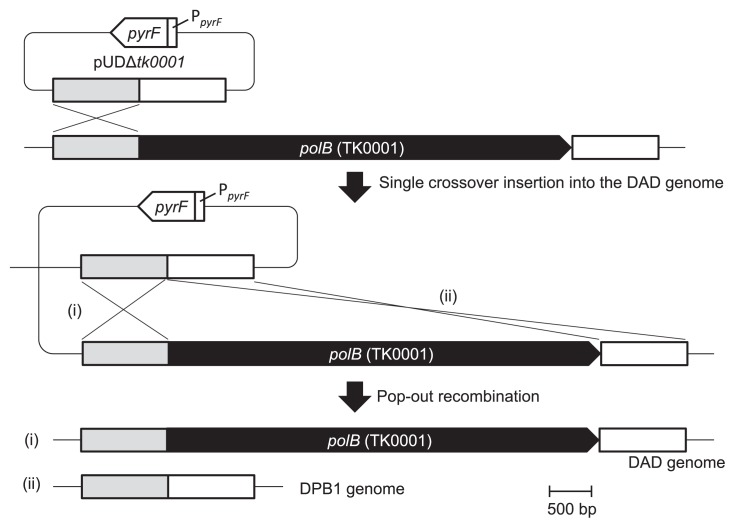
Targeted disruption of *polB* by homologous recombination in *Thermococcus kodakarensis* DAD using the plasmid pUDΔ*tk0001*. The homologous 5′ and 3′ flanking regions of *polB* (*ca*. 1.0 kbp each) are shown in gray and white, respectively. P*_pyrF_* indicates the putative promoter region of the operon containing *pyrF*. Pop-out recombination between the homologous 5′ (i) or 3′ flanking region (ii) resulted in reversion to the wild type or *polB* gene disruption, respectively.

DNA fragments containing the 5′ and 3′ flanking regions (*ca*. 1.0 kbp each) of *polB* (NCBI locus tag: TK_RS00010) were amplified from the genomic DNA of *T. kodakarensis* using the primer sets tk0001-D1/tk0001-D2 and tk0001-D3/tk0001-D4, respectively. Amplified fragments were inserted into the *Hin*cII site of pUC19. The resulting plasmids were digested with *Sph*I/*Pst*I (5′ flanking) and *Pst*I/*Eco*RI (3′ flanking) and inserted into pUC19 digested with *Sph*I and *Eco*RI, and DNA fragments digested with *Sph*I and *Eco*RI were then inserted into the gene disruption plasmid pUD3 containing the *pyrF* marker gene cassette (45). The resultant plasmid was designated pUDΔ*tk0001* (Fig. 1). The theoretical background for gene disruption by homologous recombination has been described previously (37).

The disruption of *polB* in *T. kodakarensis* DAD was performed as described previously (27) with slight modifications. Cells were cultivated for 17 h in ASW-YT-S^0^ medium supplemented with 0.5 mM agmatine sulfate (*T. kodakarensis* DAD is an agmatine auxotroph because it lacks *pdaD*) (9). Cells were harvested and resuspended in 200 μL of 0.8×ASW and kept on ice for 30 min. After the addition of 12 μg of pUDΔ*tk0001* to the cell suspension and a further incubation on ice for 1 h, treated cells were cultivated in ASW-AA-S^0^ medium with 0.5 mM agmatine sulfate at 85°C for 24 h to enrich transformants that displayed uracil prototrophy due to the integration of the plasmid into the chromosome by single-crossover recombination. After the incubation, cells were diluted with 0.8×ASW and spread onto solid ASW-YT medium supplemented with 0.5 mM agmatine sulfate, 0.75% (w/v) 5-fluoroorotic acid (5-FOA), and 45 mM NaOH, and then incubated at 85°C for 2 d. Since cells with an intact pyrimidine biosynthetic pathway convert 5-FOA to 5-fluoroorotidine-5′-phosphate, which is toxic, only *pyrF*-deficient cells via the second pop-out recombination grew under these conditions. Transformants were selected and their genotype was examined by a polymerase chain reaction (PCR) with the primer set tk0001-D1/tk0001DSEQ4-2 and DNA sequencing. The constructed Δ*polB* strain was designated DPB1.

### Southern blot analysis

Genomic DNA (20 μg) from strain DAD or DPB1 was digested with *Pvu*II and subjected to 1% agarose gel electrophoresis. Nucleic acids were transferred to a nylon membrane (Hybond™-N^+^; GE Healthcare, Chicago, IL, USA) by semi-dry electroblotting. A DIG High Prime DNA Labeling and Detection Starter Kit I (for color detection with NBT/BCIP) (Roche Diagnostics, Rotkreuz, Switzerland) was used for labeling and detection.

To construct *polB* and *polB*-upstream probes, a DNA fragment encoding the entire *polB* gene was amplified from the genomic DNA of *T. kodakarensis* using the primer set tk0001-F2/tk0001-R and then cloned into pUC19 digested with *Hin*cII. The resulting plasmid was purified from *E. coli* DH5α and digested with *Sph*I/*Bam*HI; the DNA fragment obtained was used as the *polB* probe. The plasmid containing the fragment amplified with the primer sets tk0001-D1/tk0001-D2, as mentioned previously, was digested with *Sph*I/*Pst*I (5′ flanking); the DNA fragment obtained was used as the *polB-*upstream probe.

### Western blot analysis

Cell extracts from strains in the stationary or exponential growth phase were subjected to SDS-PAGE. After electroblotting onto a polyvinylidene difluoride membrane, the proteins on the membrane were reacted with anti-TkoPol B, anti-PfuDP1, and anti-TkoPrimase antisera, which were prepared by immunizing rabbits with the recombinant proteins *T. kodakarensis* Pol B, *P. furiosus* Pol D (small subunit), and *T. kodakarensis* DNA primase, respectively. Regarding the detection of Pol B, Pol D, and DNA primase, anti-Rabbit IgG HRP (Rabbit TrueBlot; Rockland Immunochemicals, PA, USA) was used as the secondary antibody. Proteins were visualized with an enhanced chemiluminescence detection system (Merck Millipore, Burlington, MA, USA) and an LAS-3000 image analyzer (FUJIFILM, Tokyo, Japan). Regarding the detection of Pol B in *polB*-complemented cells, an anti-Rabbit IgG (Fc) AP-Conjugate (Promega, Madison, WI, USA) was used as the secondary antibody together with nitro blue tetrazolium and 5-bromo-4-chloro-3-indolyl phosphate.

### DNA polymerase activity assay of cell-free extracts

Strains DAD and DPB1 were cultivated in ASW-YT-Pyr medium supplemented with 0.5 mM agmatine sulfate at 85°C for 9 h until they had reached an optical density of 0.35 at a wavelength of 660 nm. Harvested cells (2×10^10^ cells) were suspended in 1.5 mL of buffer A (50 mM Tris-HCl [pH 8.0], 1 mM ethylenediaminetetraacetic acid, 0.5 mM dithiothreitol, 10% glycerol) and disrupted by sonication. The supernatant was applied to an anion-exchange column (HiTrap Q HP, 1 mL; GE Healthcare), and the chromatograph was developed with a 0 to 1.0 M NaCl gradient in 10 mL of buffer A.

A nucleotide incorporation assay was used to detect the DNA polymerizing activity of chromatographic fractions. The reaction mixtures (20 μL) contained 20 mM Tris-HCl [pH 8.8], 5 mM MgCl_2_, 1 mM dithiothreitol, 0.2 mg mL^−1^ of activated calf thymus DNA, 10 μM deoxynucleoside triphosphate containing 200 nM thymidine 5′-[methyl-^3^H] triphosphate, and 2 μL of the cell fraction. The reaction mixture was incubated at 74°C for 5 min and acidinsoluble radioactivity bound to a DE81 filter was detected with a scintillation counter.

### Construction of the plasmid for the complementation analysis of strain DPB1

We constructed the plasmid pUC-PTgdh to add the promoter (P*_gdh_*) and terminator (T*_gdh_*) regions of the gene encoding glutamate dehydrogenase (*gdh*) into *polB*. The intergenic region (*ca*. 0.6 kbp) containing P*_gdh_* between the coding region of *gdh* (TK1431) and the adjacent gene TK1432 was amplified from the genomic DNA of *T. kodakarensis* by PCR with the primer set Pgdh-F/Pgdh-R, and the PCR product was used as the template for PCR with the primer set Pgdh-F/Pgdh-R2. A DNA fragment (*ca*. 0.1 kbp) containing T*_gdh_* was amplified from the genomic DNA of *T. kodakarensis* by PCR with the primer set Tgdh-F2/Tgdh-R, and the PCR product was used as the template for PCR with the primer set PTgdh-S/Tgdh-R. Since the amplified P*_gdh_* and T*_gdh_* regions shared a segment with an identical sequence, the two fragments were joined by overlap extension PCR with the primer set Pgdh-F/Tgdh-R. The joined fragment was inserted into pUC19 digested with *Hin*cII, and amplified from the resulting plasmid with the primer set Pgdh-F/Tgdh-R. The unique *Nde*I recognition sequence, CATATG, in pUC19 was changed to CAAATG by inverse PCR using the primer set puc19NdeIF/puc19NdeIR, and the resulting plasmid was used as the template for inverse PCR with the primer set pucEcoRIX/pucHindIIIX to remove the multiple cloning site. The amplified fragment containing P*_gdh_* and T*_gdh_* was phosphorylated and ligated to the PCR product using the primer set puc19NdeIF/puc19NdeIR. The resulting plasmid was designated pUC-PTgdh and contained recognition sites for the restriction enzymes *Nde*I, *Nhe*I, *Bam*HI, *Eco*RI, and *Xho*I between the P*_gdh_* and T*_gdh_* regions.

A DNA fragment encoding *polB* (Δ*intein-1*, Δ*intein-2*) was amplified from the plasmid pET-*pol*(mature) (38) by PCR with the primer set Tk-polB-F/Tk-polB-R and inserted into pUC19 digested with *Hin*cII. The *polB* (Δ*intein-1*, Δ*intein-2*) fragment digested with *Nde*I/*Bam*HI was inserted into the plasmid pUC-PTgdh digested with *Nde*I/*Bam*HI. A DNA fragment containing P*_gdh_*, *polB* (Δ*intein-1*, Δ*intein-2*), and T*_gdh_* was obtained by digestion with *Eco*RV and inserted into the *E. coli*–*T. kodakarensis* shuttle plasmid pTKG02 digested with *Eco*RV. The resulting plasmid was designated pTKG-polB.

### Drop dilution assay

Strain DAD and its derivatives were cultured in ASW-YT-S^0^ medium supplemented with or without agmatine sulfate (0.5 mM) at 85°C for 17 h.

To elucidate the sensitivity of the strains to DNA damage by γ-rays, the incubated cells were harvested and resuspended in 0.8×ASW at a concentration of 1×10^6^ cells μL^−1^, and then irradiated with γ-rays from a ^60^Co-source at dose rates of 1 or 1.5 kGy h^−1^ at the Takasaki Advanced Radiation Research Institute (Gunma, Japan). The cell suspension was kept on ice before and after irradiation.

To elucidate the sensitivity of the strains to agents that induce DNA alkylation and interstrand cross-linking, the incubated cells were resuspended in 0.8×ASW supplemented with methyl methanesulfonate (MMS) or mitomycin C (MMC), respectively, at a concentration of 1×10^6^ cells μL^−1^ and incubated at room temperature for 4 h. Cell suspensions after the treatment with γ-rays, MMS, or MMC were then serially diluted 10-fold with 0.8×ASW and spotted on solid ASW-YT medium with or without agmatine sulfate (0.5 mM).

To elucidate the sensitivity to DNA damage caused by ultraviolet C (UV-C), incubated cells were harvested and resuspended in 0.8×ASW at a concentration of 1×10^6^ cells μL^−1^. Serially diluted cells (2×10^6^, 2×10^5^, 2×10^4^, 2×10^3^, and 2×10^2^ cells) were spotted on solid ASW-YT medium, and irradiated with a germicidal ultraviolet lamp (wavelength, 254 nm; irradiance, 0.4 W m^−2^, as calculated using an UVX Radiometer; UVP, Upland, CA, USA).

After an incubation at 85°C for 2 d, living cells were transferred to polyvinylidene difluoride membranes and visualized by staining with Coomassie Brilliant Blue R-250.

## Results

### Construction of a polB deletion mutant

**Fig. 2:**
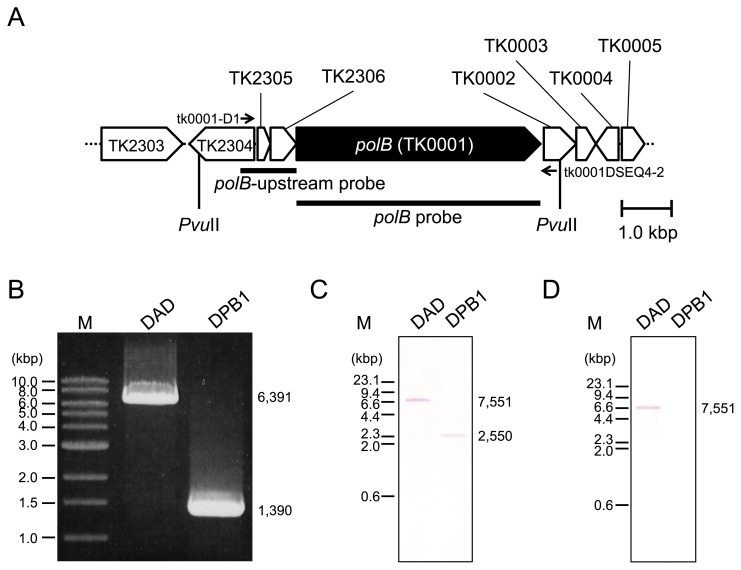
Characterization of our *polB* disruptant by a polymerase chain reaction (PCR) analysis and Southern blotting. (A) Organization of the *polB* gene locus. The *polB* gene is shown in black. The PCR primers for the genome analysis of the parental strain (DAD) and the *polB* disruptant (DPB1) are indicated by small arrows. The *polB*-upstream and *polB* probes used for the Southern blot analysis are indicated by black bars. (B) PCR analysis of the *polB* gene locus using the genomic DNAs of strains DAD and DPB1. M denotes the DNA size marker (1 kbp DNA Ladder; New England Biolabs, Ipswich, MA, USA). Confirmation of *polB* disruption by a Southern blot analysis using the *polB*-upstream probe (C) and *polB* probe (D) with the genomic DNAs of strains DAD and DPB1 digested with *Pvu*II. M denotes the DNA size marker (*Hin*dIII-digested λ DNA). The expected number of base pairs in each signal calculated from the genome sequence of *T. kodakarensis* is shown to the right of each panel.

To examine the physiological role of Pol B in *T. kodakarensis*, a *polB* deletion mutant was constructed. The plasmid pUDΔ*tk0001* harboring *pyrF* as a selectable marker and the 5′ and 3′ flanking regions of *polB* was constructed and introduced into the chromosomal DNA of strain DAD by single-crossover recombination (Fig. 1). Uracil-prototrophic cells, due to the integration of the plasmid pUDΔ*tk0001*, were concentrated in ASW-AA-S^0^ medium without uracil. 5-FOA-resistant cells generated by the excision of the *pyrF* marker gene after popout recombination were then isolated on solid ASW-YT medium containing 0.75% (w/v) 5-FOA. The mutant genotype was confirmed by a PCR analysis with the primer set tk0001-D1/tk0001DSEQ4-2, which annealed outside the *polB* encoding region (Fig. 2A). A 1,390-bp DNA fragment corresponding to the Δ*polB* locus formed by pop-out recombination was observed, indicating that *polB* was deleted (Fig. 2B). The resulting Δ*polB* mutant was designated strain DPB1.

The deletion of *polB* in DPB1 was further confirmed by Southern blotting and a PCR analysis. Southern blotting with a *polB*-upstream probe revealed a decrease in the length of the *Pvu*II fragment (from 7,551 to 2,550 bp) due to the deletion of *polB* (Fig. 2C), and Southern blotting with the *polB* probe revealed the absence of *polB* in the chromosome of DPB1 cells (Fig. 2D).

**Fig. 3:**
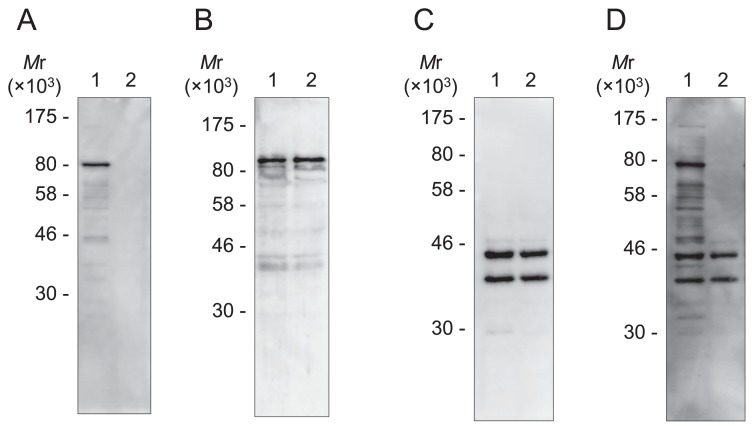
Detection of Pol B, Pol D, and DNA primase in *Thermococcus kodakarensis* cells by Western blotting. *T. kodakarensis* DAD (lane 1) and DPB1 (lane 2) cells in the exponential growth phase were harvested, and whole cell extracts from 1×10^7^ cells (A, D), 4×10^7^ cells (B), and 2×10^7^ cells (C) were subjected to 10% SDS-PAGE followed by a Western blot analysis using anti-TkoPol B (A), anti-PfuDP1 (B), anti-TkoPrimase antisera (C), and both anti-TkoPol B and anti-TkoPrimase antisera (D). After the detection of Pol B (A), the same membrane was subjected to anti-TkoPrimase antiserum as the blotting control (D). Prestained protein markers were obtained from New England Biolabs (Ipswich, MA, USA).

The absence of Pol B in strain DPB1 was confirmed by Western blotting (Fig. 3A). Pol D and DNA primase were detected with comparable signal intensities in strains DAD and DPB1 (Fig. 3B and C). In addition, after the detection of Pol B using anti-TkoPol B antiserum (Fig. 3A), the same membrane was exposed to anti-TkoPrimase antiserum as a blotting control (Fig. 3D) and Pol B was not detected in strain DPB1, whereas DNA primase was detected in DAD and DPB1 at similar signal intensities.

**Fig. 4:**
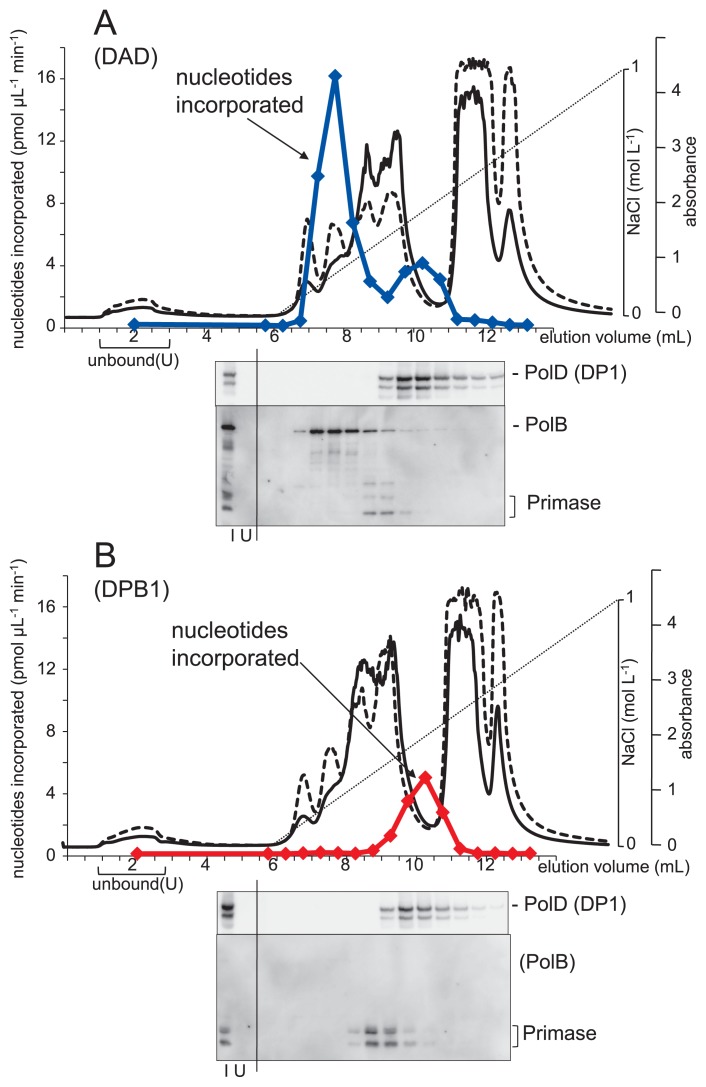
Cell extracts of *Thermococcus kodakarensis* DAD (A) and DPB1 (B) were fractionated by anion exchange chromatography. The eluted proteins were monitored by measuring absorbance at 280 nm (solid lines) and 260 nm (broken lines). DNA polymerase activity was measured by a nucleotide incorporation assay at 74°C for 5 min using a reaction mixture containing thymidine 5′-[methyl-^3^H] triphosphate (colored lines). The linear dotted line in the elution profiles indicates the NaCl concentration gradient from 0 to 1 M. Fractions were subjected to Western blotting using the antisera indicated to the right of each panel. Representative gel images are shown under the fractions. I, input fraction; U, unbound fraction.

Gefter *et al*. discovered *E. coli* DNA polymerase III, a replicative DNA polymerase, in chromatographic fractions of a *polA*^−^ cell extract (12). Therefore, to check for the existence of novel DNA polymerases in strains DAD and DPB1, cell extracts were fractionated by anion exchange chromatography and subjected to a nucleotide incorporation assay (Fig. 4). In strain DAD, major DNA polymerizing activity was detected in fractions containing Pol B, while minor DNA polymerizing activity was detected in fractions containing Pol D and DNA primase (Fig. 4A). In contrast, in strain DPB1, DNA polymerizing activity was only detected in fractions containing Pol D and DNA primase (Fig. 4B). In *T. kodakarensis*, Pol D and DNA primase may both synthesize DNA (11), although the activity of DNA primase is markedly lower than that of Pol D (17).

### Construction of a polB-complemented strain

**Fig. 5:**
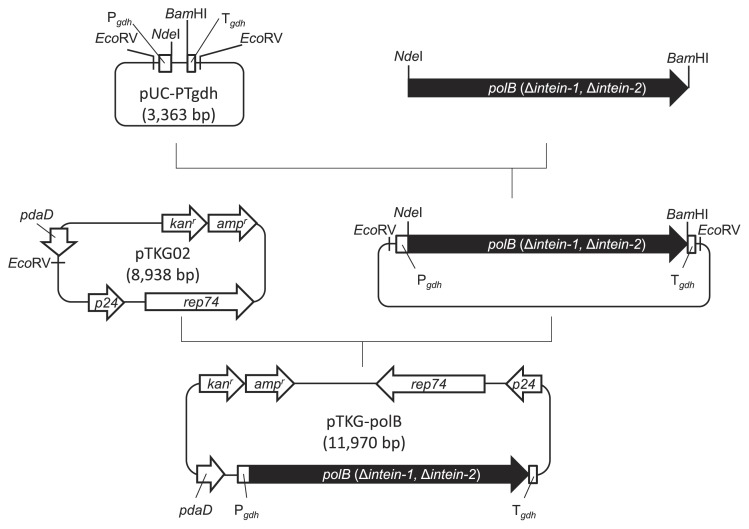
Construction of the plasmid pTKG-polB for the *polB*-complementation analysis. pTKG02 is a derivative plasmid of pTK02, as previously described by Nagaoka *et al*. (27). The DNA fragment digested with *Nde*I and *Bam*HI encoding a mature form of Pol B, which did not contain intervening sequences (*intein-1* and *intein-2*), was inserted into pUC-PTgdh digested with *Nde*I and *Bam*HI. P*_gdh_* and T*_gdh_* indicate the promoter and terminator, respectively, of *gdh*, which encodes glutamate dehydrogenase. The resulting plasmid was digested with *Eco*RV and the DNA fragment was then inserted into pTKG02 digested with *Eco*RV.

To express the Pol B protein in DPB1 cells, the promoter and terminator of *gdh* were used because the *gdh* promoter leads to strong constitutive gene expression in *T. kodakarensis* irrespective of culture conditions (*e.g*., media and growth temperature) (27, 33). Plasmid construction is summarized in Fig. 5.

The plasmid, pUC-PTgdh, containing a promoter-terminator cassette with recognition sites for the restriction enzymes *Nde*I, *Nhe*I, *Bam*HI, *Eco*RI, and *Xho*I was constructed. *polB* in the chromosome DNA of *T. kodakarensis* contains two intervening sequences, *intein-1 and intein-2*, which are spliced out at the protein level in an autocatalytic manner. After excision of the intervening proteins, PI-*Pko*I and PI-*Pko*II, corresponding to *intein-1 and intein-2*, respectively, from the precursor protein, external protein segments were ligated to form a mature functional PolB protein. These spliced proteins, PI-*Pko*I and PI-*Pko*II, exhibit endonuclease activities at specific DNA regions (29, 38). In the gene complementation analysis, the PolB protein was produced from *polB* without any intervening sequences in DPB1 cells because the effects of the production of PI-*Pko*I and PI-*Pko*II on the viability of DPB1 cells need to be omitted. The fragment encoding *polB* without any intervening sequences was ligated with pUC-PTgdh for insertion between the promoter and terminator derived from the *gdh* gene.

The *E. coli*–*T. kodakarensis* shuttle plasmid pTKG02, a derivative of pTK02, was used for the expression of *polB* (27). pTKG02 contains *pdaD*, which encodes arginine decarboxylase, a catalyst of the synthesis of agmatine from arginine. Agmatine is essential for the growth of *T. kodakarensis* cells (9). *T. kodakarensis* strain DAD is an agmatine auxotroph that cannot grow in the absence of agmatine because it lacks *pdaD*; however, DAD cells harboring pTKG02 may grow in the absence of agmatine. The fragment containing the *gdh* promoter, *polB* (Δ*intein-1*, Δ*intein-2*), and *gdh* terminator was inserted into pTKG02 digested with *Eco*RV. The resulting plasmid was designated pTKG-polB.

**Fig. 6:**
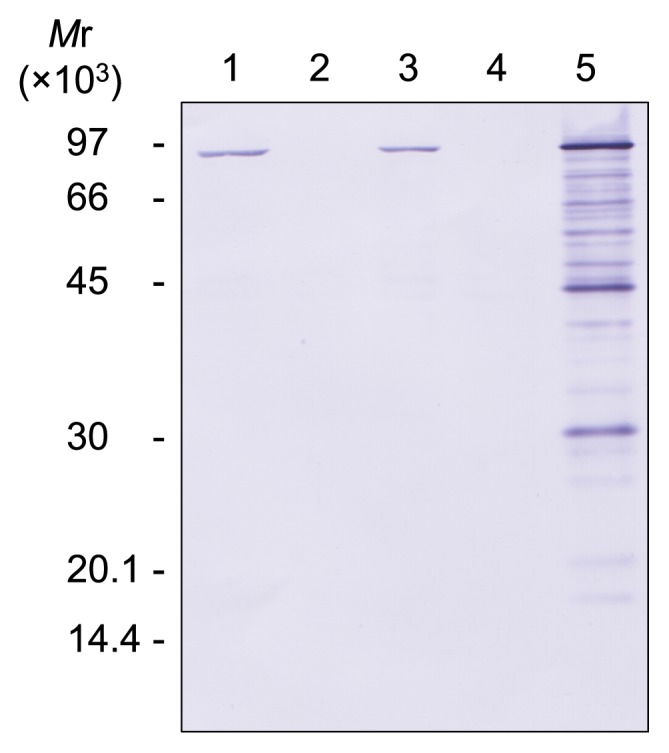
Detection of Pol B in *polB-*complemented cells by Western blotting. Whole cell extracts from 15 μg of wet cells in the stationary growth phase were subjected to 12% SDS-PAGE followed by Western blotting using anti-TkoPol B antisera. Lanes 1 and 2, strains DAD and DPB1, respectively. Lanes 3 and 4, strains DAD and DPB1 harboring the parental plasmid pTKG02, respectively. Lane 5, strain DPB1 harboring pTKG-polB.

**Fig. 7:**
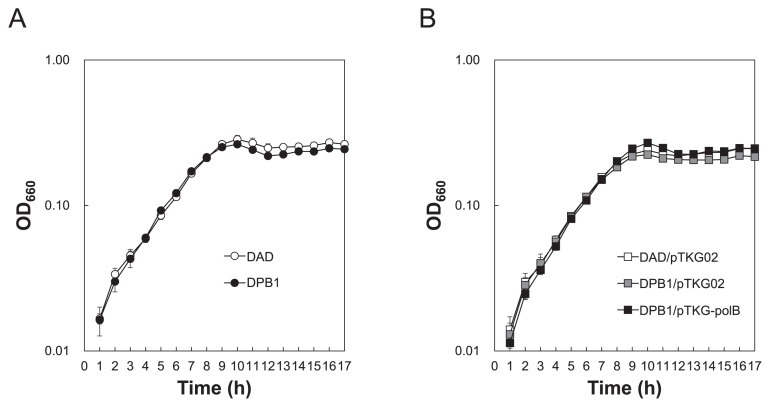
Growth characteristics of *polB* disruptants and complementants at 85°C. (A) Growth curves of *Thermococcus kodakarensis* DAD and DPB1 grown in ASW-YT-S^0^ medium with agmatine. (B) Growth curves of DAD harboring pTKG02, DPB1 harboring pTKG02, and DPB1 harboring pTKG-polB grown in ASW-YT-S^0^ medium without agmatine. OD_660_, optical density measured at a wavelength of 660 nm. Error bars represent the standard errors from triplicate growth monitoring.

After the transformation of strain DPB1 with pTKG-polB, Western blotting was performed to confirm the expression of Pol B (Fig. 6). Pol B expression was detected in the *polB-*complemented strain (DPB1 harboring pTKG-polB) and in DAD with or without pTKG02, whereas it was not detected in DPB1 with or without pTKG02. The amount of Pol B expressed by the *polB*-complemented strain was higher than that expressed by DAD with or without pTKG02. Signals corresponding to the proteolytic degradation of Pol B were found in *polB*-complemented cells; however, DPB1 with or without pTKG-polB grew normally under the same conditions, as reported by Čuboňová *et al.* (6) (Fig. 7). These results indicated that the lack or overexpression of Pol B does not affect cell growth in *T. kodakarensis*.

### Sensitivity of the polB disruptants and complementants to DNA damage

**Fig. 8:**
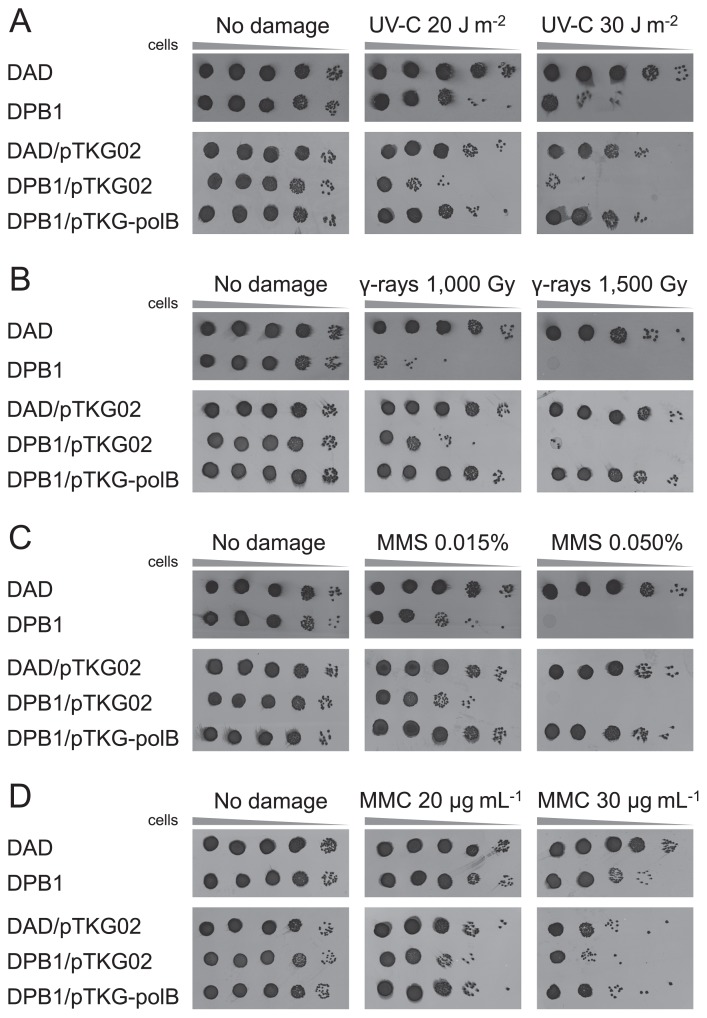
Sensitivities of *polB* disruptants and complementants to DNA damage caused by exposure to ultraviolet C (UV-C) irradiation (A), γ-ray irradiation (B), methyl methanesulfonate (MMS) (C), or mitomycin C (MMC) (D). A drop dilution assay was performed using DAD, DPB1, DAD harboring pTKG02 (DAD/pTKG02), DPB1 harboring pTKG02 (DPB1/pTKG02), and DPB1 harboring pTKG-polB (DPB1/pTKG-polB). Serially diluted cells (2×10^6^, 2×10^5^, 2×10^4^, 2×10^3^, and 2×10^2^ cells, from left to right) were spotted on ASW-YT plates before or after the treatment. After an incubation at 85°C for 2 d, living cells were transferred to polyvinylidene difluoride membranes and visualized by staining with Coomassie Brilliant Blue R-250.

A drop dilution assay was performed to evaluate the sensitivity of the strains to DNA damage caused by exposure to UV-C, γ-rays, MMS, or MMC (Fig. 8).

When untreated, possession of the *E. coli*–*T. kodakarensis* shuttle plasmid pTKG02 did not affect cell viability. In addition, DPB1 with or without pTKG-polB showed the same cell viability as the parental strain, DAD. This result suggested that the lack or overexpression of Pol B did not affect cell viability in *T. kodakarensis*.

When exposed to UV-C irradiation, which mainly induces the formation of intrastrand cross-links that results in the production of cyclobutane pyrimidine dimers and pyrimidine–pyrimidine (6-4) photoproducts, the strains not harboring *polB* (*i.e*., DPB1 and DPB1/pTKG02) showed higher sensitivity than those harboring *polB* ( *i.e*., DAD, DAD/pTKG02, and DPB1/pTKG-polB) (Fig. 8A). DAD harboring pTKG02 showed higher sensitivity than DAD not harboring pTKG02, indicating that pTKG02 decreased the viability of cells exposed to UV-C irradiation. When exposed to MMC which induces the formation of interstrand cross-links in double-stranded DNA and inhibits replication fork progression, the strains harboring the plasmid pTKG02 showed higher sensitivity than when they were exposed to UV-C irradiation (Fig. 8D).

When exposed to γ-ray irradiation, which induces the formation of double-strand breaks, or to MMS, which introduces methylated bases (*e.g*., *N*^7^-methylguanine, *N*^3^-methyladenine, and *O*^6^-methylguanine) in DNA strands, DPB1 showed higher sensitivity than DAD, and the introduction of pTKG-polB to DPB1 restored resistance to the wild-type level (Fig. 8B and C).

Overall, DPB1 was more sensitive than DAD to all of the treatments and the presence of pTKG-polB in DPB1 restored resistance to DNA damage-related stress to an equivalent level to that of DAD harboring pTKG02. This result indicated that the observed sensitivity to DNA damage-related stress was due to the loss of *polB*.

## Discussion

*T. kodakarensis* expresses two DNA polymerases, Pol B and Pol D. We herein successfully disrupted *polB* in the *T. kodakarensis* genome to generate a mutant, designated DPB1. DPB1 showed higher sensitivity to all of the tested DNA-damaging treatments (UV-C, MMS, MMC, and γ-rays) than the parental strain, DAD (Fig. 8). UV-C induces the formation of cyclobutane pyrimidine dimers and pyrimidine-pyrimidine (6-4) photoproducts in chromosomal DNA. These lesions block the progression of DNA synthesis by family A, B, C, D, or X DNA polymerases, although several enzymes belonging to family Y are able to bypass these lesions (30, 44); no gene encoding a family Y DNA polymerase has yet been reported in the *T. kodakarensis* genome (10). The present results also indicated that novel DNA polymerase activity was not found in strain DPB or DAD (Fig. 4). UV-induced lesions may be repaired either by light-dependent DNA repair enzymes called DNA photolyases or via the light-independent nucleotide excision repair (NER) pathway (20). In *T. kodakarensis*, UV-induced lesions appear to be repaired via the NER system because the *T. kodakarensis* genome does not contain any genes that encode DNA photolyases (10), and the disruption of genes associated with the NER pathway produces strains that are more sensitive to UV irradiation than their parental strains (8).

MMS introduces methylated bases into DNA strands, and MMC induces the formation of interstrand cross-links in double-stranded DNA and inhibits replication fork progression. This DNA damage appears to be repaired via the NER pathway in *T. kodakarensis* because the deletion of genes involved in the NER pathway produces strains that are more sensitive to these agents than their parental strains (8). In the present study, when strains were exposed to UV-C or MMC, DAD harboring pTKG02 showed higher sensitivity than DAD not harboring the plasmid. MMC is used for plasmid curing because it inhibits DNA replication and represses transcription due to the formation of interstrand cross-links in double-stranded DNA (40). Irradiation with UV-C also induces interstrand cross-links in double-stranded DNA (25). Therefore, a possible explanation for the higher sensitivities of the pTKG02-harboring strain to exposure to UV-C and MMC is the direct inhibition of plasmid replication by the formation of interstrand cross-links due to stalling replication forks and the indirect inhibition of plasmid replication through the repression of gene transcription related to plasmid replication on pTKG02, both of which cause the loss of plasmids in cells. Therefore, plasmid-cured cells no longer grow on a plate without agmatine.

γ-Ray irradiation induces double-strand breaks in DNA. Double-strand breaks in chromosomal DNA are considered to be repaired via the homologous recombination pathway. The sensitivity of DPB1 to DNA damage caused by UV-C, MMS, MMC, and γ-rays was suppressed by the introduction of a plasmid harboring *polB* into cells, suggesting that the exposure-sensitive phenotype of strain DPB1 was due to the loss of *polB*. In *Thermococcus*, Pol B exhibits strong strand-displacement activity and the ability to perform gap filling (13), which suggests that gap filling and strand-displacement synthesis by Pol B are important parts of the NER and homologous recombination pathways.

The growth of strain DPB1 was similar with that of the parental strain, DAD (Fig. 7). This is consistent with recently published findings (6, 36). The absence of Pol B did not affect cell growth, suggesting that Pol D is responsible for the synthesis of both the leading and lagging strands in DPB1 cells.

The RNA segment of Okazaki fragments must be removed for ligation to the preceding Okazaki fragment. Removal of the RNA segment at the 5′-terminus of Okazaki fragments was previously suggested to be accomplished by Fen1, RNase HII, or GAN (4). Fen1 is a DNA endonuclease specific for flap-structured DNA made by strand displacement in the RNA primer region of the former Okazaki fragment. However, the strand-displacement activity of Pol D is markedly weaker than that of Pol B. *In vitro* analyses revealed that the majority of lagging strand synthesis by Pol D with PCNA, RFC, Fen1, and DNA ligase was stopped before Okazaki fragment maturation (13, 15); therefore, it currently remains unclear whether the strand-displacement activity of Pol D is sufficient for Okazaki fragment processing in DPB1 cells. GAN forms a complex with Pol D *in vivo* and degrades single-stranded DNA in the 5′ → 3′ direction (22). However, *in vitro* experiments have shown that its activity is inhibited under metal ion concentrations that mimic those in *T. kodakarensis* (28). Furthermore, GAN cannot degrade RNA *in vitro* (22). In contrast to these findings, gene disruption experiments have suggested that GAN participates in the removal of the RNA segment of the Okazaki fragment (4). Even though RNase HII cleaves the RNA/DNA junction of Okazaki fragments, a single ribonucleotide remains at the 5′-terminus of the DNA segment (14). GAN may degrade the RNA segment of Okazaki fragments by associating with the replisome in *T. kodakarensis*.

The present study revealed that Pol B is important for DNA repair, not DNA replication. Further analyses are needed to fully elucidate the mechanisms underlying DNA replication in *T. kodakarensis*. However, the present study suggests that Pol D has the ability to synthesize both the leading and lagging DNA strands in *T. kodakarensis*; this result is consistent with previous findings (6). In the high temperature environments in which *Thermococcales* members live, cells are exposed to chemical stresses, such as the deamination of cytosines and adenines, depurination, the oxidation of guanines, and the methylation of bases and phosphates. DNA double-strand breaks are induced by heat as well as ionizing radiation. These DNA-damaging reactions are accelerated at elevated temperatures (23). Hence, hyperthermophiles have unique active and efficient repair systems to maintain their genomic integrity (19). The present results suggest that the function of Pol B is essential for these repair systems, which allow for survival in harsh environments.

## Figures and Tables

**Table 1:**
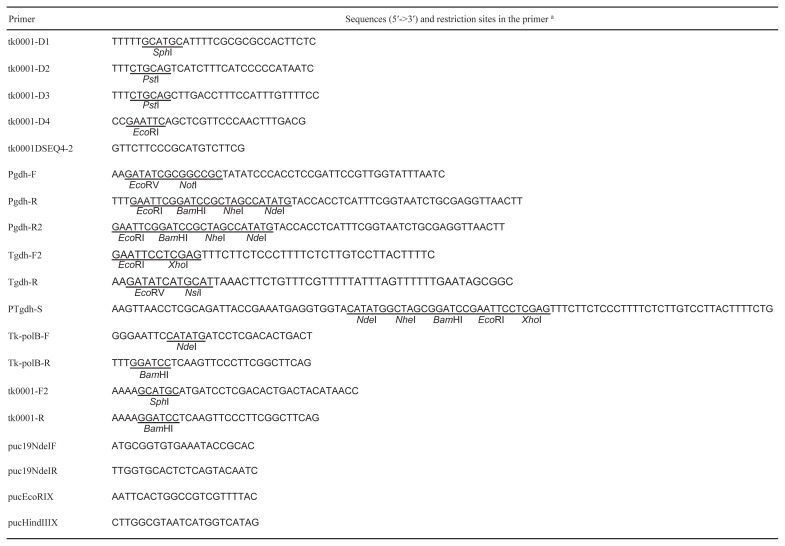
Primers used in the present study.

a The restrictions sites are underlined.
